# Editorial: Music Therapy in Geriatrics

**DOI:** 10.3389/fmed.2020.00087

**Published:** 2020-03-24

**Authors:** Suzanne B. Hanser, Amy Clements-Cortes, Melissa Mercadal-Brotons, Concetta Maria Tomaino

**Affiliations:** ^1^Department of Music Therapy, Berklee College of Music, Boston, MA, United States; ^2^Department of Music, Wilfrid Laurier University, Waterloo, ON, Canada; ^3^Department of Music Education, Escola Superior de Música de Catalunya, Barcelona, Spain; ^4^Institute for Music and Neurologic Function, Mount Vernon, NY, United States

**Keywords:** music, music therapy (MT), geriatrics, depression, Alzheimer's disease, intergenerational, caregivers, dementia

The World Health Organization (WHO) reports that the population of people living in the world who are over 60 years of age will almost double between 2015 and 2050, going from 12% of the world's inhabitants to 22%. WHO points out that by 2020, the number of people over 60 will be in excess of the number who are younger than 5 years old. Meanwhile, those over the age of 85 will continue to increase drastically. Projections estimate that the number of people of advanced age, beyond 80 years, will increase from 1950 to 2050 by 26% (1).

These rapidly changing demographics have led to major concerns regarding the growing burden and costs to healthcare systems worldwide. Services may be required for assistance with activities of daily living for community-dwelling elders and for long-term care for those who lose their independence or require more extensive treatment. These and other challenges of advanced age emphasize the need for creative interventions that delay long-term care placement and maintain older adults in their homes and communities. Non-pharmacological remedies have obvious advantages, and are being sought to manage complex medical needs of older adults. Innovative, cost-effective approaches are required to ward off isolation and enhance wellness and wellbeing.

WHO has also established dementia as a public health priority and designed a global action plan to address the problems faced by individuals with this condition as well as their caregivers. This organization estimates that there will be 82 million people with dementia by 2030 and 152 people living with this condition by 2050, and an increasing number will reside in countries where low and middle incomes are most prevalent.

Fortunately, there is a growing body of evidence to support the outcomes of music therapy interventions. This special issue of *Frontiers in Medicine* on *Music Therapy in Geriatrics* addresses several ways in which music therapy is providing creative, non-pharmacological, cost-effective programming, both in the long-term care setting and in the community https://www.frontiersin.org/research-topics/6858/music-therapy-in-geriatrics#articles. The contributions to this issue represent a diversity of clinical approaches to music therapy. First, a description of potential mechanisms underlying responses to music therapy in geriatrics is foundational in its interpretation of current research and knowledge. A distance-based intergenerational project extols the benefits of “virtual exchanges” between young and old. Other articles describe the impact of various music therapy strategies on many challenges faced by individuals with dementia and their families: (1) how depression may be reduced while general wellbeing is enhanced in the nursing home setting; (2) how communication between individuals with dementia and their spouses in the nursing home can be improved; (3) how “remini-sing” can affect persons with dementia and their family caregivers in the community; (4) how singing affects quality of life and both positive and negative affect; (5) how therapeutic songwriting aids family caregivers and their loved ones in day care, and (6) how people with Alzheimer's disease and their partners can benefit from a music therapy support group in the community.

Clements-Cortes and Bartel present a four-level model of the mechanisms of music response to help understand current music therapy approaches in geriatric care. The levels relate to learned cognitive responses, cognitive activation of neural circuits, stimulated neural coherence, and cellular and genetic changes to specific frequencies. Each level is illustrated with research and directions for research collaborations between neuroscientists, neurologists and music therapists.

Belgrave and Keown examined the use of virtual exchange technologies to investigate potential cross age benefits and preconceived ideas between younger and older individuals participating in an intergenerational virtual music project. Encouraging results point to improved cross age interactions and attitudes, while supporting the foundational basis for the potential of music therapy interventions to build relationships using virtual and live interactions.

Ray and Götell demonstrate the positive effects of 2-week music therapy interventions followed by 2-week music-based caregiving activities led by CNAs, on the depression level of nursing home residents with moderate to severe dementia. In addition to a significant decrease in depression after the 2-week of music therapy sessions, depression stabilized post-music-based interventions, suggesting that music therapy, and music-based activities may improve wellbeing in nursing home residents with dementia.

Dassa addresses the challenges of family caregivers in communicating with their loved ones who have dementia. An individualized database of personal music and photographs provided a source of positive shared memories, while improving connection.

Tamplin et al. present the findings of a 20-week therapeutic group singing intervention (Remini-Sing) for people with dementia and family caregiver dyads on the relationship quality; life and caregiver satisfaction, flourishing, and depression for caregivers; and anxiety, apathy, agitation, and quality of life for people with dementia. Although no statistically significant changes were observed from pre- to post-intervention, favorable relationship quality and wellbeing measures were sustained over the intervention.

Cho presents a study assessing quality of life and participant affect by comparing a music therapy singing group, a music medicine listening group, and a TV control group. Fifty-two participants' results indicate that the music therapy group singing condition improved affect and participants' quality of life.

Baker et al. aimed to test the feasibility of implementing a group songwriting program with family caregivers of people living with dementia. Fourteen caregivers participated in the songwriting group or control condition. Quantitative assessments included measures of depression (PHQ-9), perceptions of caregiver experience (PACQ), and perceptions of their relationship of caregiving experience (QCPR). Qualitative data suggested that coping may be a more relevant construct to measure than caregiver-patient relationship quality or caregivers' perception of caregiving.

Rio explains how individuals with AD may experience identity loss, and caregivers may experience physical and emotional stressors. An evidence-based music therapy program catering to preferences, abilities, and cultural factors for individuals with dementia and their caregivers demonstrated that music therapy was effective in reducing caregiver strain, while providing opportunities for emotional support and connection.

Taken together, these articles demonstrate how diverse music therapy and music-based interventions can affect health and wellbeing in the geriatric population.

## Author Contributions

SH wrote the introductory remarks. AC-C, MM-B, and CT contributed short reviews of the articles.

### Conflict of Interest

The authors declare that the research was conducted in the absence of any commercial or financial relationships that could be construed as a potential conflict of interest.
